# Traumatic brain injury and anger proneness: results from the Atherosclerosis Risk in Communities (ARIC) study

**DOI:** 10.3389/fpsyg.2025.1546443

**Published:** 2025-06-25

**Authors:** Connor A. Law, Holly Elser, Alexa E. Walter, Thomas H. Mosley, Keenan Walker, Rebecca F. Gottesman, Andrea L. C. Schneider

**Affiliations:** ^1^Department of Neurology, Perelman School of Medicine at the University of Pennsylvania, Philadelphia, PA, United States; ^2^Hospital of the University of Pennsylvania, Philadelphia, PA, United States; ^3^The MIND Center, University of Mississippi Medical Center, Jackson, MS, United States; ^4^National Institute of Aging Intramural Research Program, Baltimore, MD, United States; ^5^National Institute of Neurological Disorders and Stroke Intramural Research Program, Bethesda, MD, United States; ^6^Department of Biostatistics, Epidemiology, and Informatics, Perelman School of Medicine at the University of Pennsylvania, Philadelphia, PA, United States

**Keywords:** traumatic brain injury, anger proneness, trait anger, cohort, epidemiology

## Abstract

**Background/objective:**

Associations of traumatic brain injury (TBI) with subsequent increased anger proneness have been studied in younger populations, but less is known about potential bidirectional associations between TBI and anger proneness among older populations. This study aimed to investigate bidirectional associations between anger proneness and TBI among community-dwelling participants in the Atherosclerosis Risk in Communities Study.

**Methods:**

TBI was defined by self-report and ICD-9/10 codes. Anger proneness was defined using the Spielberger Trait Anger Scale. We performed 3 analyses: cross-sectional associations of prior TBI with anger proneness (Visit 2, 1990–1992, *N* = 13,694), associations of interval TBI with change in anger proneness (Visit 2, 1990–1992 to Visit 4, 1996–1998, *N* = 9,022), and prospective associations of baseline anger proneness with incident TBI (Visit 2, 1990–1992 to 12/31/2020, *N* = 11,713). Adjusted Tobit, linear, and Cox-proportional hazards regression models estimated associations, respectively.

**Results:**

Overall, participants were a mean age of 57 years at Visit 2, 55% were female, and 24% were Black. In cross-sectional analyses, prior TBI was associated with slightly higher anger proneness (*β* = 0.35, 95% CI = 0.17, 0.54). In change analyses, interval TBI was not significantly associated with change in anger proneness score over time (*β* = 0.16, 95% CI = −0.16, 0.48). In prospective analyses, increasing baseline anger proneness was not significantly associated with incident TBI (moderate anger proneness: HR = 1.05, 95% CI = 0.95, 1.15; high anger proneness: HR = 1.15, 95% CI = 0.97, 1.37).

**Conclusion:**

In conclusion, this study did not find evidence for associations between TBI and anger proneness in this older population. Further research regarding relationships between anger proneness and TBI may not be warranted in older populations.

## Introduction

Traumatic brain injury (TBI) is associated with significant short- and long-term morbidity and mortality (Schneider et al., 2021; Schneider et al., 2018). In particular, TBI is associated with cognitive (i.e., memory and executive functioning impairments) (Haarbauer-Krupa et al., 2021; Howlett et al., 2022), psychiatric (i.e., depression and post-traumatic stress disorder) (Challakere Ramaswamy et al., 2023), and personality-related (i.e., impulsivity and affective instability) (Challakere Ramaswamy et al., 2023) sequelae.

There is also evidence that TBI can change an individual’s propensity for anger (Murphy et al., 2022). In the past half century, anger has been conceptualized using the state–trait model, which splits the concept into *state anger*–the transitory emotional continuum of being annoyed, irritated, or otherwise angered–and *trait anger*, which is defined by an individual’s more stable temperamental vulnerability or proneness to becoming angered (Richard et al., 2022). While anger has historically been conceptualized as a personality trait that remains relatively static over the lifecourse (Chida and Steptoe, 2009), more recent research suggests that there are effective interventions, such as cognitive behavioral therapy and training avoidance tendencies towards threatening situations, which can be used to decrease anger proneness (Veenstra et al., 2018). Anger proneness, or trait anger, has been associated with multiple negative health outcomes (Veenstra et al., 2018), including coronary heart disease (Chida and Steptoe, 2009), diabetes (Golden et al., 2006), and stroke (Williams et al., 2002). Behaviorally, high anger proneness is associated with increased risk-taking behaviors (Deffenbacher et al., 2003) and impulsivity (Richard et al., 2022), as well as an impairment in effortful control (Wilkowski and Robinson, 2010). Additionally, high anger proneness is associated with an increased frequency in motor vehicle crashes (Deffenbacher et al., 2003), intimate partner violence, and other types of assault (Stith et al., 2004) all of which are common mechanisms for TBI (Centers for Disease Control and Prevention, National Center for Injury Prevention and Control, 2023).

While prior literature has reported increases in anger proneness and other personality changes following TBI (Kim et al., 1999; Tateno et al., 2003; Baguley et al., 2006; Rao et al., 2009), less work has investigated anger proneness as a risk factor for TBI, particularly among middle-age and older individuals. Indeed, the highest incidence of TBI occurs among older individuals, with unintentional falls being the most common mechanism of injury in this age group, followed by motor vehicle crashes (Centers for Disease Control and Prevention, 2022; Centers for Disease Control and Prevention, 2021). Leveraging data from participants in the Atherosclerosis Risk in Communities (ARIC) Study collected both before and after the occurrence of TBIs, the objective of the present study is to examine the bidirectional associations between TBI and anger proneness among community dwelling middle-aged and older adults. We hypothesized that prior TBI would be associated with greater anger proneness at baseline, that interval TBI would be associated with a greater increase in anger proneness over time, and that greater anger proneness at baseline would be associated with increased risk of incident TBI.

## Methods

### Study population

The ARIC Study is a prospective cohort of community-dwelling individuals. Participants were recruited by probability sampling of the following three communities, resulting in individuals of mainly self-reported White racial identity from Washington County, Maryland and selected suburbs of Minneapolis, Minnesota, and of mainly self-reported White and Black racial identity from Forsyth County, North Carolina. In the fourth community (Jackson, Mississippi), only individuals of self-reported Black racial identity were recruited. Participants were enrolled in 1987–1989 when they were 45–64 years old and have participated in subsequent follow-up visits, as well as annual (through 2011) and semi-annual (starting in 2012) telephone interviews. Hospitalization surveillance in all ARIC Study communities began in 1987 and is ongoing. Hospital records from hospitalizations occurring outside of ARIC Study communities are obtained if reported by participants during telephone interviews. In addition, linked Centers for Medicare and Medicaid Services (CMS) data were available for participants aged ≥65 years who were enrolled in fee-for-service part B from 1991 to 2018. All participants or their legally authorized representative provided written consent at each study visit and the ARIC Study was approved by institutional review boards at each participating institution.

**Figure 1:**
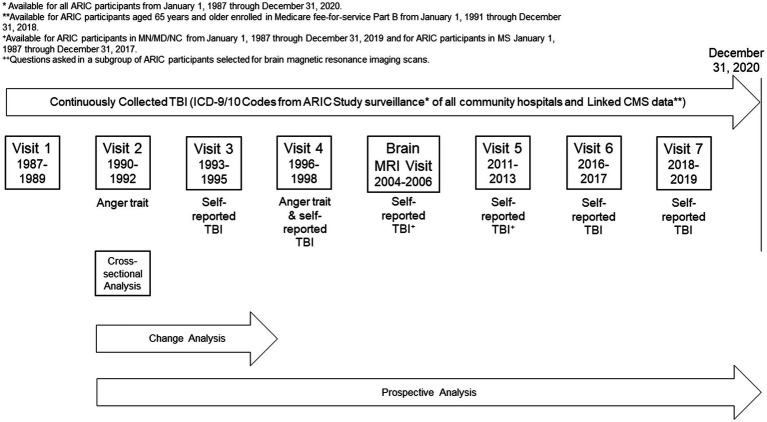
Study design. *Available for ARIC participants from January 1, 1987 through December 31, 2020. **Available for ARIC participants aged 65 years and older enrolled in Medicare fee-for-service Part B from January 1, 1991 through December 31, 2018. ^+^Questions asked in a subgroup of ARIC participants selected for brain magnetic resonance imaging scans.

**Figure 2:**
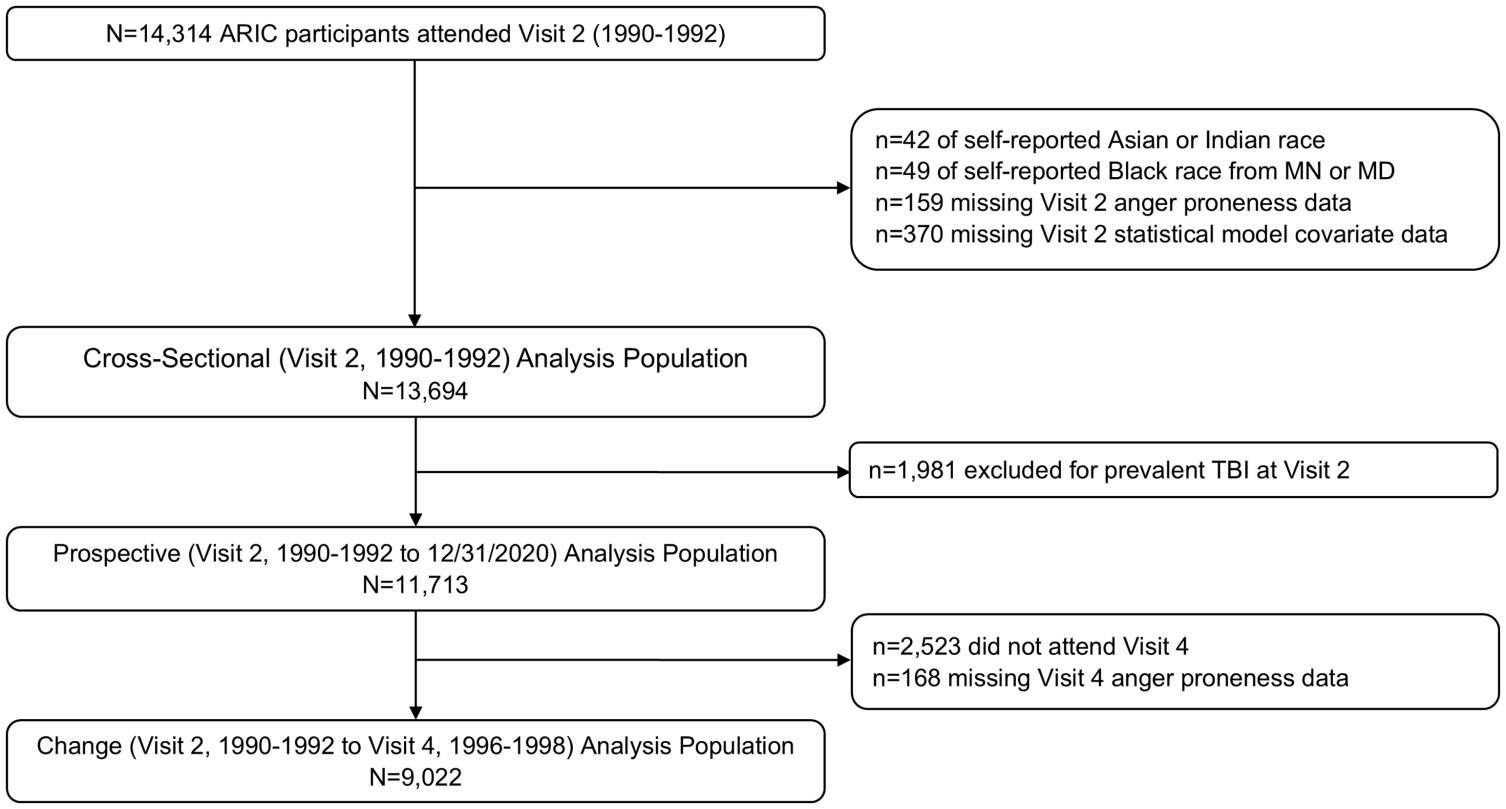
Study population definitions and inclusion/exclusion criteria.

ARIC Visit 2 (1990–1992) served as the baseline for all analyses as this was the first visit during which anger proneness was assessed (Figure 1). Of the 14,314 participants who attended Visit 2, 42 were excluded for self-identifying as Asian or American Indian racial identity, and 49 were excluded for self-identifying as Black racial identity within the Minnesota or Maryland communities due to small numbers and racial identity/center aliasing (Figure 2). Additionally, 159 were excluded for missing anger proneness data at Visit 2, and 370 were excluded for missing covariates included in statistical models. Among the 13,694 participants included in the cross-sectional analysis, 1,981 were excluded from the change and prospective and change analyses for having a prevalent TBI at Visit 2. Finally, of the 11,713 participants included in the prospective analysis, 2,523 participants were excluded from the change analysis for not attending Visit 4, with an additional 168 excluded for missing Visit 4 anger proneness data; this resulted in 9,022 participants in the change analysis population.

### TBI definition

TBI was defined based on data obtained from self-report questions asked during study visits and from diagnostic codes from the *International Classification of Diseases, Ninth/Tenth Revisions* (ICD-9/10) from ARIC Study hospitalization surveillance and linked CMS data. The self-report questions inquired about prior TBIs which required medical attention or were associated with loss of consciousness, number of prior TBIs, and year of prior injuries (Supplementary Table 1). Month and day of self-reported TBIs were imputed using the random point method (Vandormael et al., 2018). To identify diagnostic code defined head injuries, we used the Centers for Disease Control and Preventions (CDC) surveillance definition for TBI (Langlois et al., 2002; Hedegaard et al., 2016) (Supplementary Table 2).

In secondary analyses, we additionally considered the number of head injuries (no head injury; one head injury; two or more head injuries) and the severity of the head injury (among those injuries identified by ICD-9/10 codes; severity defined in accordance with the Department of Defense definition (Cené et al., 2012) as no head injury; mild head injury; moderate or severe/penetrating head injury).

### Anger proneness

Anger proneness was defined using the Spielberger Trait Anger Scale (Spielberger et al., 1983), which was administered to participants at ARIC Visits 2 (1990–1992) and 4 (1996–1998). This 10-question questionnaire uses a 4-point Likert Scale (1: almost never, 2: sometimes, 3: often, 4: almost always) to assess anger-temperament and anger-reaction, resulting in overall anger proneness scores ranging from 10 (low) to 40 (high) (Supplementary Table 3). In accordance with prior research (Golden et al., 2006), we considered anger proneness as a continuous variable and as a categorical variable (low anger proneness: score 10–14, moderate anger proneness: score 15–21, and high anger proneness: score 22–40).

### Other variables

The following covariates, which were assessed at ARIC Visit 2 (unless otherwise specified), were included in statistical models: age (derived from ARIC Visit 2 date and birth date), self-reported biological sex (female; male), self-reported racial identity/center (White Maryland; White Minnesota; White North Carolina; Black North Carolina; Black Mississippi), education (self-reported at ARIC Visit 1, less than high school; high school, GED, or vocational school; some college, college, graduate, or professional school), marital status (married; divorced or separated; never married; widowed), military veteran status (yes; no), diabetes (yes; no, defined as self-reported physician diagnosis, use of diabetes medications, fasting glucose ≥126 mg/dL, or non-fasting glucose ≥200 mg/dL), hypertension (yes; no, defined as self-reported physician diagnosis, use of hypertension medication, systolic blood pressure ≥140 mmHg, or diastolic blood pressure ≥90 mmHg), cigarette smoking (current; former; never), and alcohol consumption (current; former; never).

In sensitivity analyses, we also considered depression, defined by high vital exhaustion on the Maastricht Vital Exhaustion Questionnaire, as a potential effect modifier. The Maastricht Vital Exhaustion Questionnaire consists of 21 questions scored on a 0–2 scale (0: no, 1: do not know, 2: yes). The Maastricht Vital Exhaustion Questionnaire has previously been shown to have a 0.62 correlation with the Beck Depression Inventory (Garg et al., 2021). In accordance with prior studies (Cené et al., 2012), we defined depression (high vital exhaustion) as a score ≥14.

### Statistical analysis

All analyses were conducted using R Version 4.2.2 (R Foundation for Statistical Computing, Vienna, Austria) using two-tailed tests with statistical significance defined *a priori* as *p* < 0.05. Participant characteristics are reported by TBI and anger proneness status as means and standard deviations for continuous variables and as number and proportions for categorical variables.

We performed three analyses to evaluate bidirectional associations of TBI and anger proneness (Figure 1). First, we conducted a cross-sectional association of prior TBI with anger proneness based on data from Visit 2 (1990–1992). For these analyses, we used adjusted Tobit regression models to evaluate the association between prior TBI and anger proneness to reduce the impact of floor effects observed on the Spielberger Trait Anger Scale (Supplementary Figure 1). Second, we examined the association of interval TBI with change in anger proneness scores between Visit 2 and Visit 4. Adjusted linear regression models were used to evaluate the association between interval TBI and change in anger proneness score over time (defined as Visit 4 score minus Visit 2 score) given the normally distributed change in anger proneness data (Supplementary Figure 2). As a sensitivity analysis, we modeled change in anger proneness using a linear mixed effects model that included a random intercept and a random slope and employed an unstructured covariance matrix. Finally, we examined the association of baseline anger proneness with incident TBI measured from Visit 2 onward. These prospective analyses utilized adjusted Cox proportional hazard regression models to examine the relationship between baseline anger proneness and incident TBI. Visual inspection of the Schoenfeld residuals and complementary log–log plots confirmed that the proportional hazards assumption was met (Supplementary Figure 3). Time since ARIC Visit 2 was used as the time scale with follow-up extending to the date of first TBI, study withdrawal/loss to follow-up, death, or administrative censoring on 12/31/2020. As secondary analyses, we performed adjusted Fine and Gray proportional hazards models to account for the competing risk of death (Fine and Gray, 1999). All statistical models were adjusted for age, self-reported biological sex, self-reported racial identity/center, education, military veteran status, marital status, diabetes, hypertension, cigarette smoking, and alcohol consumption, except for secondary analyses investigating head injury severity which are presented unadjusted due to small numbers.

In secondary analyses, given prior findings on differences in TBI and anger proneness by age (Downing et al., 2024), biological sex (Gupte et al., 2019), racial identity (Maldonado et al., 2023), and depression status (Hudak et al., 2012), all analyses had these subgroups formally evaluated for multiplicative interaction. If evidence for interaction by self-reported racial identify was present, we performed a sensitivity analysis among participants from the North Carolina center to distinguish racial identity from center. Additionally, sensitivity analyses were conducted stratified by TBI definition source (self-reported TBI vs. ICD-code defined TBI).

## Results

### Cross-sectional associations between prior TBI and anger proneness

Of the 13,694 participants included in the cross-sectional analyses, 1,978 participants experienced at least one prior TBI. Overall, participants were a mean age of 57.0 years, 55.1% self-reported as biologically female, and 24.2% self-reported Black racial identity (Table 1). Compared to individuals without TBI, individuals with prior TBI were less likely to self-report as biologically female (41.9 vs. 57.3%) and self-report their racial identify as Black (14.8 vs. 25.7%) and were more likely to have greater than high school education (42.6 vs. 36.0%), be military veterans (35.0 vs. 21.1%), and be current consumers of alcohol (62.3 vs. 55.8%).

**Table 1:** Baseline participant characteristics stratified by prevalent and incident TBI status (cross-sectional analysis and change analysis populations), ARIC visit 2 (1990–1992).

	Cross-sectional analysis population		Change analysis population	
	No prevalent TBI at visit 2 (*n* = 11,716)	Prevalent TBI at visit 2 (*n* = 1,978)	No incident TBI between visits 2 and 4 (*n* = 8,632)	Incident TBI between visits 2 and 4 (*n* = 390)
Age (years), mean (SD)	57.1 (5.7)	56.4 (5.6)	56.9 (5.7)	57.3 (5.8)
Sex, *n* (%)				
Female	6,710 (57.3)	829 (41.9)	4,992 (57.8)	251 (64.4)
Male	5,006 (42.7)	1,149 (58.1)	3,640 (42.2)	139 (35.6)
Race-center, *n* (%)				
Washington County, Maryland White	2,955 (25.2)	517 (26.1)	2,294 (26.6)	122 (31.3)
Minneapolis, Minnesota White	3,111 (26.6)	606 (30.6)	2,478 (28.7)	97 (24.9)
Forsyth County, North Carolina White	2,642 (22.6)	561 (28.4)	1,918 (22.2)	79 (20.3)
Forsyth County, North Carolina Black	330 (2.8)	34 (1.7)	196 (2.3)	10 (2.6)
Jackson, Mississippi Black	2,678 (22.9)	260 (13.1)	1,746 (20.2)	82 (21.0)
Education, *n* (%)				
Less than high school	2,617 (22.3)	312 (15.8)	1,632 (18.9)	85 (21.8)
High school, GED, or vocational school	4,883 (41.7)	823 (41.6)	3,693 (42.8)	164 (42.1)
At least some college	4,216 (36.0)	843 (42.6)	3,307 (38.3)	141 (36.2)
Marital status, *n* (%)				
Married	9,264 (79.1)	1,610 (81.4)	6,995 (81.0)	310 (79.5)
Divorced or separated	1,315 (11.2)	224 (11.3)	903 (10.5)	34 (98.7)
Never married	238 (2.0)	41 (2.1)	159 (1.8)	10 (2.6)
Widowed	899 (7.7)	103 (5.2)	575 (6.7)	36 (9.2)
Military Veteran, *n* (%)	2,471 (21.1)	693 (35.0)	2,116 (24.5)	68 (17.4)
Diabetes, *n* (%)	1,795 (15.3)	265 (13.4)	1,104 (12.8)	61 (15.6)
Hypertension, *n* (%)	4,285 (36.6)	622 (31.4)	2,899 (33.6)	138 (35.4)
Cigarette smoking, *n* (%)				
Current	2,637 (22.5)	425 (21.5)	1,666 (19.3)	93 (23.8)
Former	4,355 (37.2)	852 (43.1)	3,267 (37.8)	134 (34.4)
Never	4,724 (40.3)	701 (35.4)	3,699 (42.9)	163 (41.8)
Alcohol consumption, *n* (%)				
Current	4,355 (37.2)	1,233 (62.3)	5,003 (58.0)	219 (56.2)
Former	4,724 (40.3)	426 (21.5)	1,652 (19.1)	71 (18.2)
Never	6,537 (55.8)	319 (16.1)	1,977 (22.9)	100 (25.6)
Depression, *n* (%)	3,604 (30.8)	603 (30.5)	2,428 (28.1)	135 (34.6)

When using Tobit models to account for floor effects (Supplementary Figure 1), individuals with prior TBI had anger proneness scores that were 0.35 points higher (95% CI = 0.17, 0.54) than individuals without prior TBI in fully adjusted models (Table 2). Prior TBI was associated with higher anger proneness score among individuals of self-reported White race (*β* = 0.40; 95% CI = 0.21, 0.60) but not among individuals of self-reported Black race (*β* = 0.03; 95% CI = −0.48, 0.55), *p*-interaction = 0.03. In order to distinguish self-reported racial identify from center, we performed a sensitivity analysis among North Carolina center participants and found similar results to our main analysis (White: *β* = 0.57; 95% CI = 0.22, 0.92 and Black: *β* = 0.03; 95% CI = 0.03; −0.48, 0.55). There was no evidence of interaction by age, self-reported biological sex, or depression status. In stratified sensitivity analyses, associations of self-reported TBI and ICD-code defined TBIs with anger proneness were consistent with the primary analysis (Supplementary Table 4).

**Table 2:** Associations of prevalent TBI with anger proneness score (cross-sectional ARIC visit 2, 1990–1992) and of incident TBI with change in anger trait (ARIC visit 2, 1990–1992 to ARIC visit 4, 1996–1998).

	Cross-sectional analyses* anger proneness score *β* (95% CI)		Cross-sectional analyses* anger proneness score standardized *β* (95% CI)		Change analyses** change in anger proneness score *β* (95% CI)		Change analyses** Change in anger proneness score standardized *β* (95% CI)	
	No prevalent TBI at visit 2	Prevalent TBI at visit 2	No prevalent TBI at visit 2	Prevalent TBI at visit 2	No incident TBI between visits 2 and 4	Incident TBI between visits 2 and 4	No Incident TBI between visits 2 and 4	Incident TBI between visits 2 and 4
Overall	0 (Reference)	0.35 (0.17, 0.54)	0 (Reference)	0.09 (0.04, 0.14)	0 (Reference)	0.16 (−0.16, 0.48)	0 (Reference)	0.05 (−0.05, 0.15)
Stratified by median baseline age								
Baseline Age <57 years	0 (Reference)	0.47 (0.21, 0.73)	0 (Reference)	0.12 (0.06, 0.19)	0 (Reference)	−0.08 (−0.54, 0.39)	0 (Reference)	−0.02 (−0.17, 0.12)
Baseline age ≥57 years	0 (Reference)	0.26 (−0.01, 0.53)	0 (Reference)	0.07 (0.00, 0.14)	0 (Reference)	0.36 (−0.08, 0.80)	0 (Reference)	0.11 (−0.03, 0.25)
Stratified by sex								
Male	0 (Reference)	0.44 (0.18, 0.70)	0 (Reference)	0.12 (0.05, 0.19)	0 (Reference)	−0.15 (−0.69, 0.39)	0 (Reference)	−0.05 (−0.22, 0.12)
Female	0 (Reference)	0.22 (−0.05, 0.49)	0 (Reference)	0.06 (−0.01, 0.13)	0 (Reference)	0.34 (−0.05, 0.74)	0 (Reference)	0.11 (−0.02, 0.23)
Stratified by race								
White	0 (Reference)	0.40 (0.21, 0.60)	0 (Reference)	0.11 (0.06, 0.16)	0 (Reference)	0.06 (−0.29, 0.40)	0 (Reference)	0.02 (−0.09, 0.13)
Black	0 (Reference)	0.03 (−0.48, 0.55)	0 (Reference)	0.01 (−0.13, 0.15)	0 (Reference)	0.54 (−0.25, 1.32)	0 (Reference)	0.17 (−0.08, 0.42)
Stratified by depression status								
No depression	0 (Reference)	0.35 (0.15, 0.55)	0 (Reference)	0.09 (0.04, 0.15)	0 (Reference)	0.26 (−0.11, 0.62)	0 (Reference)	0.08 (−0.04, 0.20)
Depression	0 (Reference)	−0.04 (−0.40, 0.33)	0 (Reference)	−0.01 (−0.11, 0.09)	0 (Reference)	0.05 (−0.56, 0.67)	0 (Reference)	0.02 (−0.18. 0.21)

*Tobit regression model. **Linear regression model. Models were adjusted for age, sex, race/center, education, military veteran status, marital status, diabetes, hypertension, cigarette smoking, and alcohol consumption. P-interaction by age = 0.23, p-interaction by sex = 0.09, p-interaction by race = 0.03, and p-interaction by depression status = 0.11 in cross-sectional analyses. All p-interaction for age, sex, race, and depression status >0.05 in change analyses.

In secondary analyses, similar associations were seen by TBI frequency (1 TBI, *n* = 1,576: *β* = 0.35, 95% CI = 0.14, 0.55 and 2 + TBIs *n* = 402: *β* = 0.37, 95% CI = −0.01, 0.76). There were 63 individuals with mild TBI and 5 with moderate or severe/penetrating TBI among the subset of TBIs identified using ICD-9/10 codes, which resulted in limited precision in associations of TBI severity with anger proneness (mild TBI: unadjusted *β* = −0.27, 95% CI = −1.25, 0.70; moderate or severe/penetrating TBI: unadjusted *β* = −1.83, 95% CI = −5.31, 1.65).

### Association between TBI and change in anger proneness over time

Among the 9,022 participants included in the change analysis, 390 individuals experienced at least one interval TBI occurring between ARIC Visit 2 (1990–1992) and Visit 4 (1996–1998). Overall, participants were a mean age of 56.9 years at baseline, 58.1% self-reported as biologically female, and 22.6% self-reported Black racial identity. Individuals with vs. without interval TBI were more likely to self-report as biologically female (64.4 vs. 57.8%) but were otherwise similar (Table 1).

Overall, interval TBI was not significantly associated with change in anger proneness score over time (*β* = 0.16, 95% CI = −0.16, 0.48) (Table 2). There was no evidence of interaction by age, self-reported biological sex, self-reported racial identity, or depression. Similarly, we did not see any evidence for associations of interval TBI number with change in anger proneness score (1 TBI, *n* = 144: *β* = −0.07, 95% CI = −0.59, 0.44; 2 + TBIs, n = 246: *β* = 0.30, 95% CI = −0.10, 0.70). By interval TBI severity (among the interval TBIs identified by ICD-9/10 codes), interval mild TBI was not significantly associated with change in anger proneness score (*n* = 126, unadjusted *β* = 0.30, 95% CI = −0.26, 0.85), while moderate or severe/penetrating TBI was associated with lower anger proneness score over time (*n* = 27, unadjusted *β* = −1.82, 95% CI = −3.01, −0.63). In sensitivity analyses, associations of interval self-reported TBI and of interval ICD-code defined TBI with change in anger proneness over time were consistent with the main change analyses (Supplementary Table 4). Similarly, in sensitivity analyses using a linear mixed effects model, results were consistent with our primary analysis (*β* = 0.14, 95% CI = −0.18, 0.45).

### Prospective association between anger proneness and incident TBI

A total of 1,961 of the 11,713 participants in the prospective analysis had an incident TBI over a median of 14.7 years (25^th^ percentile-75th percentile = 6.9–20.5) of follow-up. Overall, the mean age of included individuals was 57.1 years at baseline, 57.3% self-reported as biologically female, and 25.7% self-reported Black racial identity (Table 3). Compared to individuals with low anger proneness, individuals with high anger proneness were more likely to have less than high school education (32.1 vs. 23.6%), be current smokers (30.2 vs. 18.1%), consumers of alcohol (61.1 vs. 50.4%), and have depression (56.4 vs. 19.3%).

**Table 3:** Baseline characteristics of participants without prevalent TBI stratified by anger trait level (prospective analysis population), ARIC visit 2 (1990–1992).

	Prospective analysis population		
	Low anger trait (*N* = 4,379)	Moderate anger trait (*N* = 6,412)	High anger trait (*N* = 922)
Age (years), mean (SD)	57.5 (5.8)	56.9 (5.7)	56.8 (5.8)
Sex, n (%)			
Female	2,540 (58.0)	3,678 (57.4)	491 (53.3)
Male	1,839 (42.0)	2,734 (42.6)	431 (46.7)
Race-center, n (%)			
Washington County, Maryland White	967 (22.1)	1,727 (26.9)	260 (28.2)
Minneapolis, Minnesota White	1,175 (26.8)	1,741 (27.2)	193 (20.9)
Forsyth County, North Carolina White	996 (22.7)	1,438 (22.4)	208 (22.6)
Forsyth County, North Carolina Black	123 (2.8)	181 (2.8)	26 (2.8)
Jackson, Mississippi Black	1,118 (25.5)	1,325 (20.7)	235 (35.5)
Education, n (%)			
Less than High School	1,032 (23.6)	1,289 (20.1)	296 (32.1)
High School, GED, or Vocational School	1,781 (40.7)	2,737 (42.7)	363 (39.4)
At least some college	1,566 (35.8)	2,386 (37.2)	263 (28.5)
Marital status, *n* (%)			
Married	3,377 (77.1)	699 (10.9)	107 (11.6)
Divorced or Separated	509 (11.6)	5,174 (80.7)	711 (77.1)
Never married	97 (2.2)	116 (1.8)	25 (2.7)
Widowed	397 (9.1)	423 (6.6)	79 (8.6)
Military Veteran, *n* (%)	864 (19.7)	1,402 (21.9)	205 (22.2)
Diabetes, *n* (%)	663 (15.1)	948 (14.8)	184 (20.0)
Hypertension, *n* (%)	1,622 (37.0)	2,291 (35.7)	371 (40.2)
Cigarette smoking, *n* (%)			
Current	793 (18.1)	1,565 (24.4)	278 (30.2)
Former	1,527 (34.9)	2,444 (38.1)	384 (41.6)
Never	2,059 (47.0)	2,403 (37.5)	260 (28.2)
Alcohol consumption, *n* (%)			
Current	2,209 (50.4)	3,765 (58.7)	563 (61.1)
Former	879 (20.1)	1,346 (21.0)	220 (23.9)
Never	1,291 (29.5)	1,301 (20.3)	139 (15.1)
Depression, *n* (%)	844 (19.3)	2,240 (34.9)	520 (56.4)

**Figure 3:**
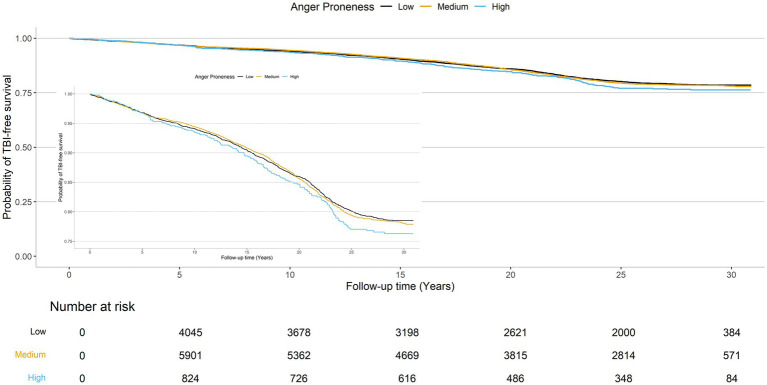
Probability of TBI-free survival.

The cumulative TBI-free survival time was comparable across baseline anger proneness levels (Figure 3). In adjusted Cox proportional hazards models increasing baseline anger proneness was not significantly associated with increased risk of incident TBI (moderate anger proneness: HR = 1.05, 95% CI = 0.95, 1.15; high anger proneness: HR = 1.15, 95% CI = 0.97, 1.37; Table 4). In models accounting for the competing risk of death, point estimates were attenuated. There was no evidence of interaction by age, self-reported biological sex, self-reported racial identify, or depression. In sensitivity analyses, associations of baseline anger proneness with incident TBI were comparable to the main analysis regardless of TBI ascertainment source (self-reported vs. ICD-code defined) (Supplementary Table 5).

**Table 4:** Adjusted hazard ratios (HRs) for the prospective associations of anger proneness with incident TBI, ARIC study visit 2, 1990–1992 through December 31, 2020.

	Low anger proneness	Moderate anger proneness	High anger proneness
Number of TBI events/person-years	729 / 91,049	1,074 / 132,825	158 / 17,878
Unadjusted IR per 1,000 PYs (95% CI)	8.01 (7.44, 8.61)	8.09 (7.61, 8.58)	8.84 (7.51, 10.33)
Cox proportional hazards model, HR (95% CI)	1 (Reference)	1.05 (0.95, 1.15)	1.15 (0.97, 1.37)
Fine-Gray proportional hazards model, HR (95% CI)	1 (Reference)	1.03 (0.94, 1.13)	1.09 (0.93, 1.29)

Models were adjusted for age, sex, race/center, education level, military veteran status, marital status, diabetes, hypertension, cigarette smoking, and alcohol consumption. All *p*-interaction for age, sex, race, and depression status >0.05.

## Discussion

Cross-sectionally we found minimal evidence that TBI was associated with increased anger proneness overall and among select subgroups with estimated differences between individuals with vs. without TBI falling below the minimum scoring increment. Changes in anger proneness were also small in magnitude. Prospective analyses suggested that anger proneness is not a significant risk factor for TBI. Taken together, the results of this study suggest that anger proneness is similar between individuals with and without TBI and is not significantly associated with incident TBI among community-dwelling middle-aged and older adults.

Our results contrast with some prior literature, which reported increases in anger proneness after TBI in select younger populations (mean age <40 years; largely clinic populations) (Arciniegas and Wortzel, 2014; Barry and Ettenhofer, 2012; Dikmen et al., 1986). Some have attributed findings of an increase in anger proneness to psychiatric sequalae and maladaptive coping mechanisms, such as substance use (Arciniegas and Wortzel, 2014; Castaño Monsalve et al., 2012), following TBI, rather than the TBIs themselves. However, some other studies have reported anger proneness as stable or decreasing over time post-injury (Arciniegas and Wortzel, 2014). Given the self-reported nature of the Spielberger Trait Anger Scale, one explanation of this is the presence of alexithymia, a condition defined by difficulty recognizing and describing a person’s own emotional state. This condition is significantly more common following TBI (Fynn et al., 2021), and it could potentially lead individuals to chronically underreport anger and/or fail to connect descriptions of anger with their own experiences. Another possible explanation is social desirability biases, whereby some individuals may be more likely to underreport anger proneness, which is generally perceived to be a negative trait.

There is a paucity of research focused on anger as a risk-factor for incident TBIs, and studies that have been performed were in select populations. One study examining anger and TBI bidirectionally found individuals with high aggression-hostility had a greater rate of incident TBIs (Matei et al., 2022). However, this study cohort consisted of 4,881 young Swiss males (mean age 25 years) and required TBI to be associated with loss of consciousness (Matei et al., 2022). Another smaller study examined TBIs among 100 men and women with diagnosed personality disorders (mean age 39 years) and found that individuals with antisocial personality disorder, which is commonly associated with aggression as a symptom, featured a notably higher rate of TBI (Hibbard et al., 2000). The differences between our study and these prior studies are likely driven by the older age of our cohort and the inclusion of individuals of both sexes, as well as a broader definition of TBI (incorporating injuries not associated with loss of consciousness).

We attempted to address the potential role of mood disorders by investigating for potential effect modification by depression status in our statistical models, although we did not find evidence for interaction. Though TBIs are heterogeneous, previous research has found that lesions in the temporal (Hibbard et al., 2000; Grafman et al., 1996) and frontal lobes (Tateno et al., 2003; Hibbard et al., 2000; Grafman et al., 1996) following TBI are associated with increased anger following TBI. Indeed, this may be in part due to mood disorders, such as depression, that may develop after TBI; selective serotonin reuptake inhibitors (SSRIs) were found to reduce post-TBI aggression (Tateno et al., 2003). Other research has suggested that repeated TBIs are linked with changes in the limbic system, which is understood to play a key role in emotion regulation (Lepage et al., 2019).

Our study is not without limitations. First, the anger proneness data was collected at only two time points and relied on self-reported data, which may be differential with respect to TBI status. Further, the sparsity of time points meant we were unable to evaluate the possibility of dynamic changes in anger proneness over time. In addition, TBI was defined by self-report and ICD codes, however prior validation studies have shown ICD code definitions of TBI to have 55–72% sensitivity and 80–85% specificity (Carlson et al., 2013; Warwick et al., 2020). Further, while the self-reported questions about TBI did change over time, they focused on TBIs requiring medical care and/or associated with loss of consciousness and therefore may not capture milder injuries that did not require medical care. Indeed, prior literature suggests that milder TBI events may be misclassified/underdiagnosed (Cota et al., 2019; Powell et al., 2008), which may have attenuated observed associations towards the null. We also did not have detailed information on clinical characteristics of the injury (e.g., post-traumatic amnesia, altered mental status), injury mechanism, or acute head imaging findings. Further, our analyses incorporating injury severity were underpowered, limiting conclusions that can be drawn. Although the ARIC Study populations are representative of the communities the participants are recruited from, our results may not generalize to younger, non-Black/non-White populations, or to other geographic regions. Our change analyses included the subset of participants with data at two visits, and these results may be impacted by selection/survivorship bias. Additionally, vital exhaustion is an imperfect proxy measure of depression; future studies with more direct measures of depression are warranted.

## Conclusion

Our results did not find strong evidence for an association between TBI and anger proneness in this community-based population of middle aged and older adults. Further research regarding relationships between anger proneness and TBI may not be warranted in older populations.

## Data Availability

The ARIC study data is publicly available. This data can be found at: NHLBI Bio LINCC (https://biolincc.nhlbi.nih.gov/home/).
