# Supplementary material for: Traumatic brain injury and anger proneness: results from the Atherosclerosis Risk in Communities (ARIC) study
Source: Front Psychol. 2025 Jun 25;16:1546443. doi: 10.3389/fpsyg.2025.1546443 (PMC12239879; doi:10.3389/fpsyg.2025.1546443)
Supplement: Supplementary file 1 [file Supplementary_file_1.docx]

Supplemental Figure 1. Distribution of Visit 2 Anger Proneness Scores.


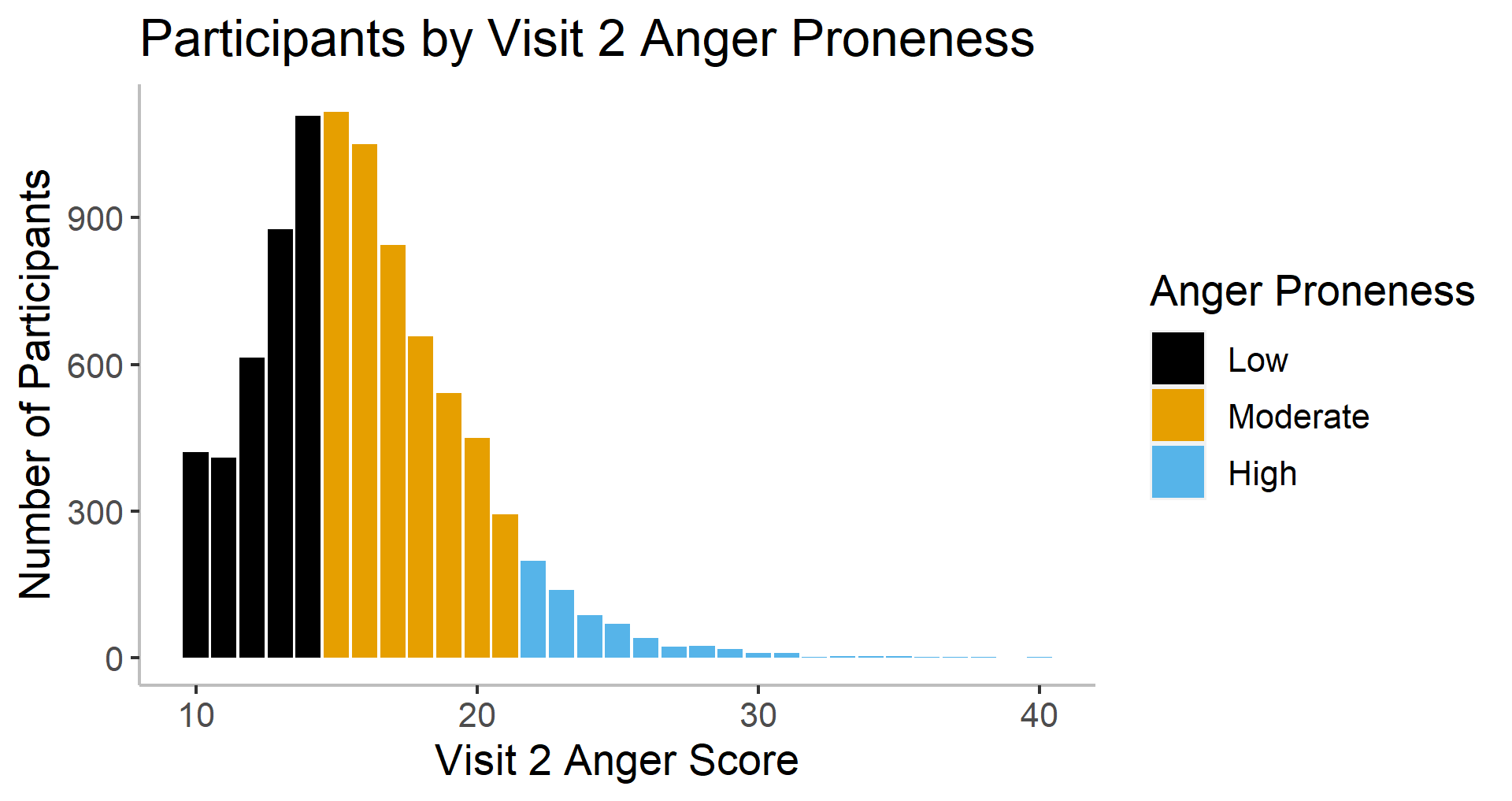


Anger proneness categories are low (10-14), moderate (15-21), and high (22-40).

Supplemental Figure 2. Distribution of Change in Anger Proneness Scores (Visit 4 – Visit 2).


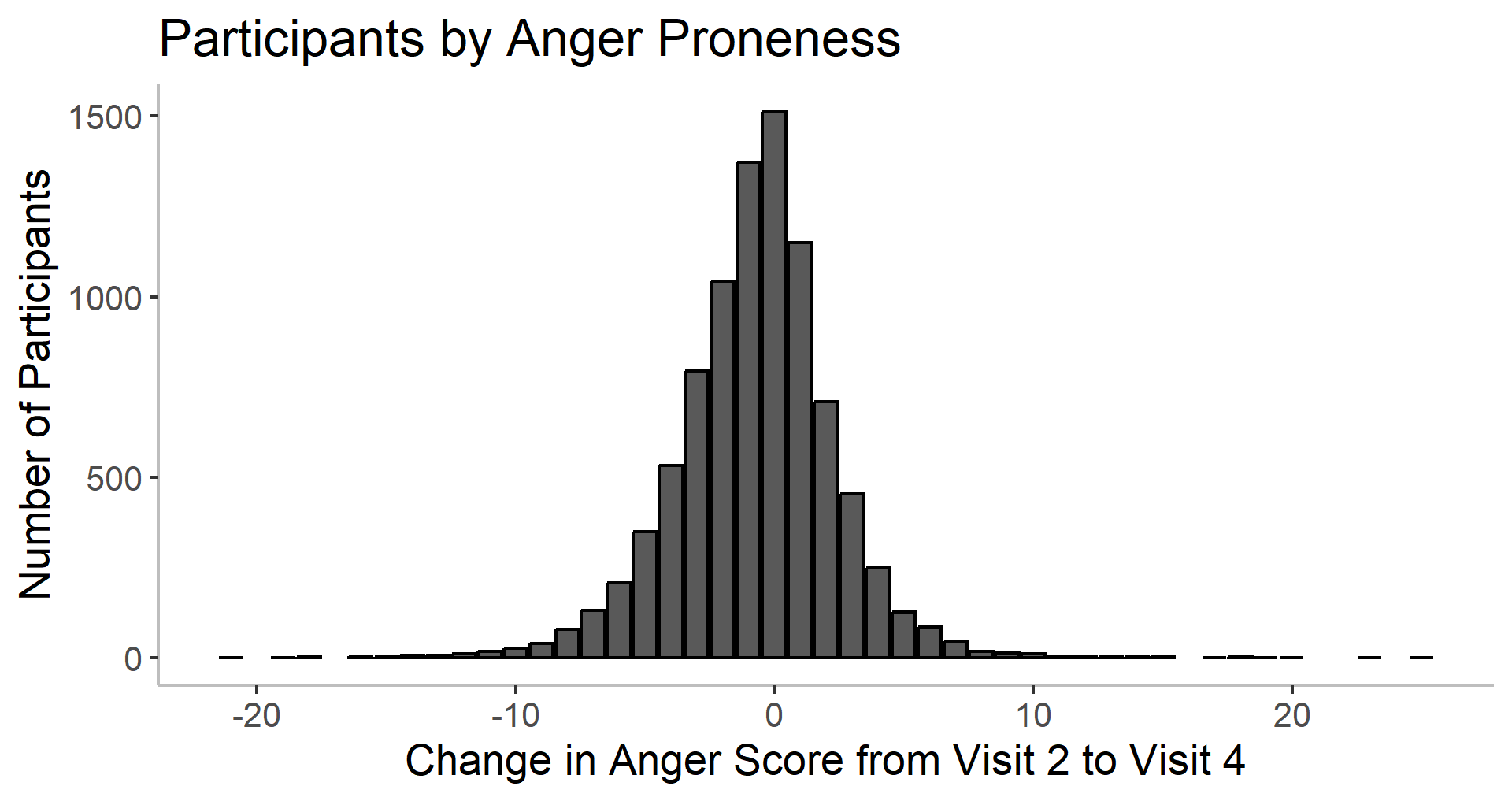


Supplemental Figure 3. Complementary Log-log Plot for Proportional Hazards Assumption.

**
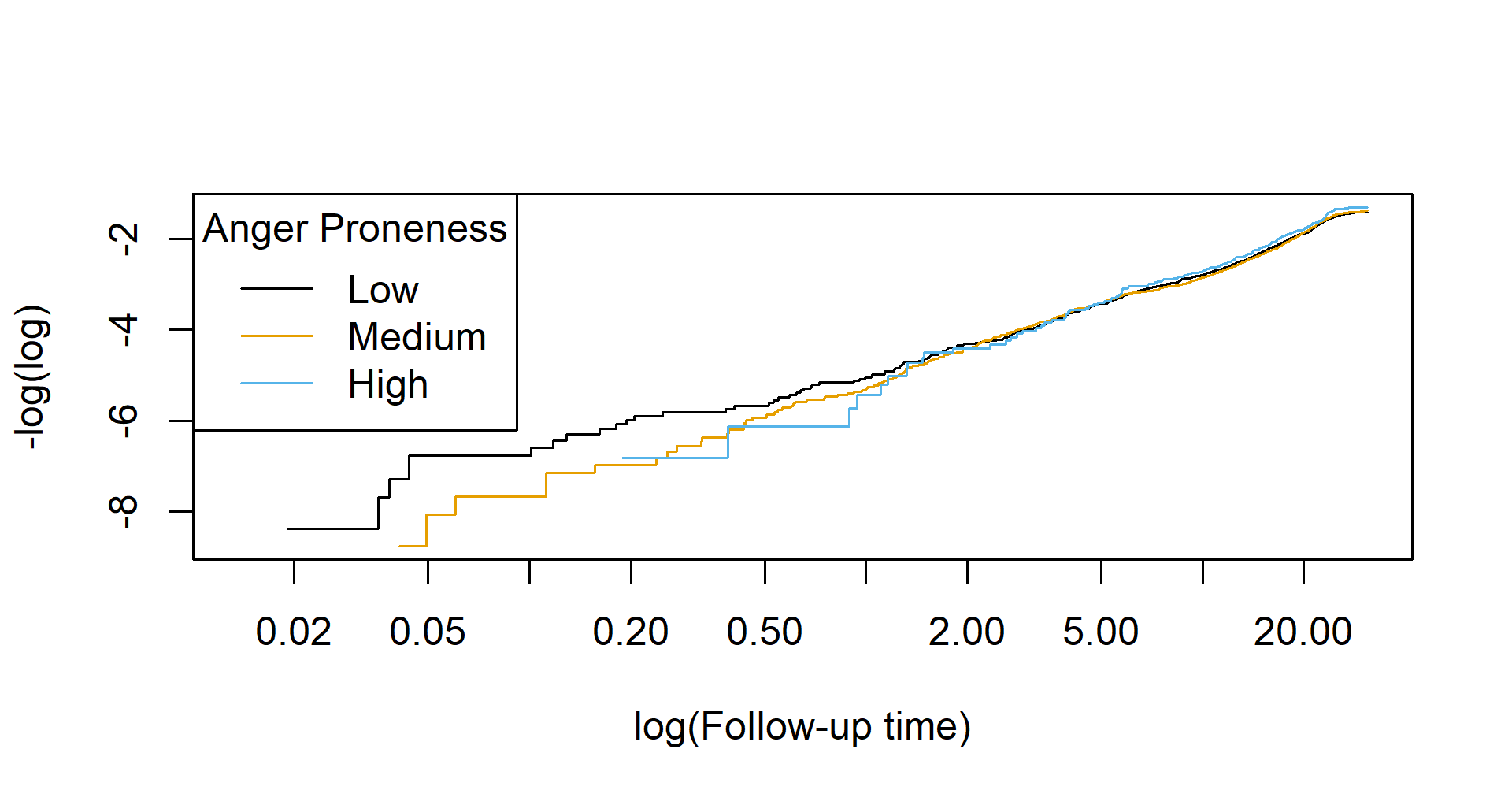
**

Supplemental Table 1. TBI Self-Report Questions.

| **ARIC Visit 3 (1993-1995)** | **ARIC Visit 4 (1996-1998)** | **ARIC Brain MRI Visit (2004-2006)*** | **ARIC Visit 5 (2011-2013)*, Visit 6 (2016-2017), and Visit 7 (2018-2019)** |
| --- | --- | --- | --- |
| Have you ever had a head injury which led you to see a physician or seek hospital care? | Have you ever had a major head injury? That is, one that resulted in your losing consciousness, no matter how briefly, or that led you to see a physician or seek hospital care? | Have you ever had a head injury that resulted in loss of consciousness (knocked out)? | Have you ever had a head injury that resulted in loss of consciousness? |
| How many times has this happened? | How many times has this happened? | How many times? | Have you had a head injury with extended loss of consciousness (>5 minutes)? |
| How many of these head injuries resulted in your losing consciousness, no matter how briefly? | How many head injuries resulted in your losing consciousness, no matter how briefly? | In what year or how old were you when this first occurred? | Have you had a head injury that resulted in long-term problems or dysfunction? |
| In what year was your head injury for which you sought medical care? | In what year was your head injury for which you lost consciousness sought medical care? | In what year or how old were you when this last occurred? |  |

*Questions asked in a subgroup of ARIC participants selected for brain magnetic resonance imagining (MRI) scans.

Supplemental Table 2. Definition of TBI by ICD-9/10 Codes.

| **ICD-9 Codes (Prior to October 2015)** | | **ICD-10 Codes (Starting October 2015)** | |
| --- | --- | --- | --- |
| 800.xx | Fracture of vault of skull | S02.0 | Fracture of vault of skull |
| 801.xx | Fracture of base of skull | S02.1X | Fracture of base of skull |
| 803.xx | Other and unqualified skull fractures | S02.8 | Fractures of other unspecified skull and facial bones |
| 804.xx | Multiple fractures involving skull or face with other bones | S02.91 | Unspecified fracture of skull |
| 850.xx | Concussion | S04.02 | Injury of optic chiasm |
| 851.xx | Cerebral laceration and contusion | S04.03X | Injury of optic tract and pathways |
| 852.xx | Subarachnoid, subdural, and extradural hemorrhage following injury | S04.04X | Injury of visual cortex |
| 853.xx | Other and unspecified intracranial hemorrhage following injury | S06.X | Intracranial injuries, concussion, traumatic cerebral edema, diffuse and focal traumatic brain injury, traumatic epidural, subdural, and subarachnoid hemorrhage |
| 854.xx | Intracranial injury of other and unspecified nature | S07.1 | Crushing injury of skull |
| 959.01 | Head injury, unspecified |  |  |

Supplemental Table 3. Spielberger Trait Anger Scale Items.

| Administered at ARIC Visit 2 (1990-1992) and ARIC Visit 4 (1996-1998) |
| --- |
| 1. I am quick tempered. |
| 2. I have a fiery temper. |
| 3. I am a hotheaded person. |
| 4. I get angry when I am slowed down by others’ mistakes. |
| 5. I feel annoyed when I am not given recognition for good work. |
| 6. I fly off the handle. |
| 7. When I get angry, I say nasty things. |
| 8. It makes me furious when I am criticized in front of others. |
| 9. When I get frustrated, I feel like hitting someone. |
| 10. I feel infuriated when I do a good job and get a poor evaluation. |

Questions are scored on a 0-4 Likert scale (Almost Never; Sometimes; Often; Almost Always).

Supplemental Table 4. Associations of Prevalent TBI with Anger Proneness Score (Cross-sectional ARIC Visit 2, 1990-1992) and of Incident TBI with Change in Anger Trait (ARIC Visit 2, 1990-1992 to ARIC Visit 4, 1996-1998), Stratified by Self-reported versus ICD-code defined TBI.

|  | Cross-Sectional Analyses*  Anger Proneness Score β (95% CI) | | Cross-Sectional Analyses*  Anger Proneness Score Standardized β (95% CI) | | Change Analyses**  Change in Anger Proneness Score β (95% CI) | | Change Analyses**  Change in Anger Proneness Score Standardized β (95% CI) | |
| --- | --- | --- | --- | --- | --- | --- | --- | --- |
|  | No Prevalent TBI at Visit 2 | Prevalent TBI at Visit 2 | No Prevalent TBI at Visit 2 | Prevalent TBI at Visit 2 | No Incident TBI Between Visits 2 and 4 | Incident TBI Between Visits 2 and 4 | No Incident TBI Between Visits 2 and 4 | Incident TBI Between Visits 2 and 4 |
| Self-reported Only | 0  (Reference) | 0.37  (0.18, 0.56) | 0 (Reference) | 0.10  (0.05, 0.15) | 0  (Reference) | 0.30  (-0.09, 0.68) | 0  (Reference) | 0.09  (-0.03, 0.22) |
| ICD-code Defined Only | 0  (Reference) | -0.07  (-0.99, 0.86) | 0 (Reference) | -0.02  (-0.26, 0.23) | 0  (Reference) | -0.09  (-0.59, 0.41) | 0  (Reference) | -0.03  (-0.19, 0.13) |

*Tobit regression model
**Linear regression model

Models were adjusted for age, sex, race/center, education, military veteran status, marital status, diabetes, hypertension, cigarette smoking, and alcohol consumption.

Supplemental Table 5. Adjusted Hazard Ratios (HRs) for the Prospective Associations of Anger Proneness with Incident TBI, ARIC Study Visit 2, 1990-1992 through December 31, 2020, Stratified by Self-reported versus ICD-code defined TBI.

|  | Low Anger Proneness | Moderate Anger Proneness | High Anger Proneness |
| --- | --- | --- | --- |
| Number of TBI Events / Person-years |  |  |  |
| Self-reported Only | 105 / 97,098 | 149 / 139,882 | 29 / 18,727 |
| ICD-code Defined Only | 645 / 92,852 | 976 / 135,205 | 134 / 18,364 |
| Unadjusted IR per 1000 PYs (95% CI) |  |  |  |
| Self-reported Only | 1.09 (0.89, 1.32) | 1.07 (0.90, 1.25) | 1.55 (1.04, 2.22) |
| ICD-code Defined Only | 6.95 (6.42, 7.50) | 7.22 (6.77, 7.69) | 7.30 (6.11, 8.64) |
| Cox proportional hazards model, HR (95% CI) |  |  |  |
| Self-reported Only | 1 (Reference) | 0.95 (0.74, 1.22) | 1.26 (0.83, 1.91) |
| ICD-code Defined Only | 1 (Reference) | 1.09 (0.99, 1.21) | 1.12 (0.93, 1.35) |
| Fine-Gray proportional hazards model, HR (95% CI) |  |  |  |
| Self-reported Only | 1 (Reference) | 0.95 (0.73, 1.22) | 1.24 (0.82, 1.87) |
| ICD-code Defined Only | 1 (Reference) | 1.07 (0.97, 1.18) | 1.06 (0.88, 1.27) |

Models were adjusted for age, sex, race/center, education level, military veteran status, marital status, diabetes, hypertension, cigarette smoking, and alcohol consumption.
